# Ventriculografia: Quando Escolher Realizá-la?

**DOI:** 10.36660/abc.20220137

**Published:** 2022-05-04

**Authors:** Gabriella Cunha Lima

**Affiliations:** 1 Faculdade de Medicina Nova Esperança João Pessoa PB Brasil Faculdade de Medicina Nova Esperança, João Pessoa, PB – Brasil; 2 Departamento de Cardiologia Intervencionista Hospital Alberto Urquiza Wanderley João Pessoa PB Brasil Departamento de Cardiologia Intervencionista - Hospital Alberto Urquiza Wanderley, João Pessoa, PB – Brasil

**Keywords:** Aterosclerose, Ecocardiografia de contraste, Angioplastia

As diretrizes atuais abordam as indicações de cateterismo cardíaco esquerdo associado à cinecoronariografia, porém a grande maioria dessas instruções não cita a ventriculografia, ficando este exame, muitas vezes, a critério do operador. A ventriculografia foi utilizada, por anos, como método padrão-ouro para função ventricular. O que este minieditorial relata são algumas considerações que tentam preencher esse vazio.^1^

No que diz a respeito à ventriculografia, podem ocorrer algumas complicações, como a nefropatia induzida por contraste (NIC), que ocorre em, aproximadamente, 1% dos pacientes sem fatores predisponentes e, em 10% a 30% daqueles com fatores de risco, embolização, arritmias e tamponamento cardíaco, e uma maior exposição à radiação.^2^

É necessário que a ventriculografia seja feita com boa qualidade e, para isso, alguns pontos precisam ser elencados, como as injeções realizadas de forma manual, a quantidade de pressão ou até mesmo injeções manuais que podem até ser perigosas se realizadas por meio de cateter com único orifício final. Além disso, os volumes de contrastes devem ser suficientes para opacificar o ventrículo e o posicionamento adequado, permitindo a quantificação automatizada e precisa dos volumes do ventrículo esquerdo e dimensões da aorta.^3^

A fração de ejeção determinada visualmente pela ventriculografia esquerda correlaciona-se de forma variável à fração de ejeção da ecocardiografia. Já em relação à ventriculografia esquerda biplano, esta se correlaciona melhor do que a ventriculografia esquerda monoplano quando comparada à ressonância magnética cardíaca (RM) para fração de ejeção, volumes ventriculares e movimentação da parede.^4^

A decisão de realizar a ventriculografia quando há outros métodos diagnósticos tem sido individualizada, variando também entre regiões geográficas e hospitais.^5^

No estudo realizado por Lima Santos et al., foram analisados prontuários de 600 pacientes submetidos à cineangiocoronariografia. Destes, 324 fizeram o exame de ventriculografia.^6^ Ademais, foram selecionados pacientes com 18 anos ou mais, atendidos em caráter de urgência ou com suspeita de DAC, e que passaram pelo procedimento da angioplastia.

No estudo, em relação às variáveis, 89.8% dos pacientes realizaram o exame no período diurno, 33,75% tinham função ventricular conhecida e 283 (47,2%) tinham síndrome coronariana crônica. Outrossim, ao ser avaliado o uso do contraste, observou-se que foram utilizados apenas 3 ml a mais, quando comparado aos pacientes que não utilizaram ventriculografia, o que chamou a atenção, visto que geralmente utiliza-se 30 ml a mais para realização do exame.

O paciente com diagnóstico de síndrome coronariana crônica tem, de maneira independente, maior chance de realizar ventriculografia. Por outro lado, ter a função ventricular esquerda conhecida, ser hipertenso, ter sido submetido à revascularização cirúrgica previa e apresentar valores aumentados de creatinina associaram-se com maior chance de não realizar a técnica.

Tais dados, até então, eram compatíveis com a literatura, porém, um achado inesperado no estudo sobreveio: a ventriculografia foi mais utilizada em pacientes com síndromes coronarianas crônicas do que naqueles com síndromes coronarianas agudas, o que, na teoria, poderia exigir uma avaliação mais imediata.

Métodos não invasivos, como as novas tecnologias desenvolvidas nos aparelhos de ecocardiografia, têm sido possíveis por meio da análise do *strain* global longitudinal (SGL) das paredes do VE, pelo diagnóstico precoce das alterações isquêmicas em pacientes com alteração de troponina, todavia, sem alteração do ECG ou ecocardiograma de repouso.^7^

Nos pacientes com choque cardiogênico, a avaliação da função ventricular pode ser prejudicada, pois o miocárdio encontra-se atordoado,^8^ sendo importante ressaltar que, nesses casos agudos, a presença de um defeito de enchimento apical sugestivo de trombo não deve ser negligenciada, pois pode ser visualizado na ventriculografia, mas não observado no eco trans torácico sem contraste.^9^

Outra necessidade no infarto com supra de ST seria avaliar complicações como ruptura aguda de parede livre, comunicação interventricular, insuficiência mitral e síndrome de Takotsubo.^10^

Na amostra, não houve nenhum paciente com complicação mecânica, portanto, essa situação não foi avaliada no estudo.

No caso de síndrome coronariana sem supra de ST, com eletrocardiograma não interpretável e doença em mais de um vaso, a ventriculografia pode ajudar na identificação da artéria culpada.^11^

Cabe mencionar que neste estudo não existe discriminação se é infarto com supra de ST ou infarto sem supra de ST.

Por fim, fica visível que o papel da ventriculografia esquerda evoluiu radicalmente ao longo do último meio século, mas recebeu pouca atenção na literatura acadêmica. É importante lembrar que a técnica e a frequência de uso da ventriculografia esquerda variam entre as regiões, instituições e a decisão do operador.

Sugerimos, então, que sejam incluídos nas diretrizes critérios para utilização do método.
