# Supplementary material for: Ventriculografia: Quando Escolher Realizá-la?
Source: Arq Bras Cardiol. 2022 May 4;118(5):974–5. [Article in Portuguese] doi: 10.36660/abc.20220137 (PMC9368883; doi:10.36660/abc.20220137)
Supplement: Translation

# Translation: Ventriculography: When to Choose to Perform It?

**Authors:** Gabriella Cunha Lima

**Affiliations:** 1 Brazil Faculdade de Medicina Nova Esperança, João Pessoa, PB – Brazil; 2 Brazil Departamento de Cardiologia Intervencionista - Hospital Alberto Urquiza Wanderley, João Pessoa, PB – Brazil

**Keywords:** Atherosclerosis, Contrast echocardiography, Angioplasty

The current guidelines address the indications for left heart catheterization associated with coronary angiography, but most of these instructions do not mention ventriculography, as this test is often at the operator’s discretion. For many years, ventriculography was used as the gold standard method for ventricular function. This mini editorial presents some insights to fill this gap.^1^

Some complications may occur concerning ventriculography, such as contrast-induced nephropathy (CIN) in approximately 1% of patients without predisposing factors and in 10% to 30% of those with risk factors, embolization, arrhythmia, cardiac tamponade, and increased radiation exposure.^2^

Ventriculography must be performed with good quality, and, for that, some points of attention need to be considered, such as manual injections, the amount of pressure or even manual injections that can be dangerous if performed using a catheter with a single end hole. Furthermore, contrast volumes must be sufficient to opacify the ventricle and properly position, allowing accurate automated quantification of left ventricular volumes and aortic dimensions.^3^

The ejection fraction visually determined by left ventriculography is variably correlated with the ejection fraction of the echocardiography. Regarding biplane left ventriculography, it correlates better than monoplane left ventriculography compared to cardiac magnetic resonance (CMR) imaging for ejection fraction, ventricular volumes, and wall motion.^4^

The decision to perform ventriculography when there are other diagnostic methods has been individualized, also varying between geographic regions and hospitals.^5^

In the study carried out by Lima Santos et al., medical records of 600 patients who underwent coronary angiography were analyzed, of which 324 underwent ventriculography.^6^ Besides, patients aged 18 years or older, treated urgently or with suspected CAD, who underwent angioplasty, were selected.

In the study, regarding the variables, 89.8% of the patients underwent the examination during the day, 33.75% had known ventricular function, and 283 (47.2%) had a chronic coronary syndrome. Furthermore, regarding the use of contrast, it was found that only 3 ml more were used, compared to patients who did not use ventriculography, which drew attention since 30 ml more is usually used to perform the test.

Patients diagnosed with chronic coronary syndrome are independently more likely to undergo ventriculography. On the other hand, having known left ventricular function, being hypertensive, having undergone coronary artery bypass grafting, and having increased creatinine levels were associated with greater chances of not having the technique performed.

Such data, until then, were consistent with those found in the literature; however, an unexpected finding emerged: ventriculography was mostly used in patients with chronic coronary syndromes than in those with acute coronary syndromes, which, in theory, could require a more immediate assessment.

Non-invasive methods, such as the new technologies developed in echocardiography devices, have been possible through global longitudinal strain (GLS) of the LV walls to diagnose early ischemic abnormalities in patients with abnormal troponin levels, however, without abnormal ECG or resting echocardiogram findings.^7^

In patients with cardiogenic shock, assessment of ventricular function may be impaired, as the myocardium is stunned.^8^ It is important to emphasize that, in these acute cases, the presence of an apical filling defect suggestive of thrombus should not be neglected, as it may be viewed on ventriculography but not on non-contrast transthoracic echo.^9^

Another requirement in ST-segment elevation infarction would be to assess complications such as acute free wall rupture, ventricular septal defect, mitral regurgitation and Takotsubo syndrome.^10^

In the sample, there were no patients with mechanical complications; therefore, this situation was not evaluated in the study.

In the case of non-ST-segment elevation coronary syndrome, with uninterpretable electrocardiogram and disease in more than one vessel, ventriculography can help identify the culprit artery.^11^

It is worth noting that there is no discrimination between ST-segment elevation infarction and non-ST-segment elevation infarction in this study.

Finally, it is apparent that the role of left ventriculography has evolved dramatically over the past half-century, but it has received little attention in the academic literature. It is important not to forget that the technique and frequency of use of left ventriculography vary between regions, institutions and according to the operator’s decision.

We, therefore, suggest that criteria for using the method be included in the guidelines.
